# Supplementary material for: A genetically encoded biosensor to monitor dynamic changes of c-di-GMP with high temporal resolution
Source: Nat Commun. 2024 May 9;15:3920. doi: 10.1038/s41467-024-48295-0 (PMC11082216; doi:10.1038/s41467-024-48295-0)
Supplement: Supplementary file 1 — Supplementary Information [file 41467_2024_48295_MOESM1_ESM.pdf]

SUPPLEMENTARY INFORMATION

A genetically encoded biosensor to monitor dynamic changes of c-di-GMP with high temporal resolution

Andreas Kaczmarczyk, Simon van Vliet, Roman Peter Jakob, Raphael Dias Teixeira, Inga Scheidat, Alberto Reinders, Alexander Klotz, Timm Maier, Urs Jenal

Biozentrum, University of Basel, 4056 Basel, Switzerland

Correspondence to: urs.jenal@unibas.ch, andreas.kaczmarczyk@unibas.ch

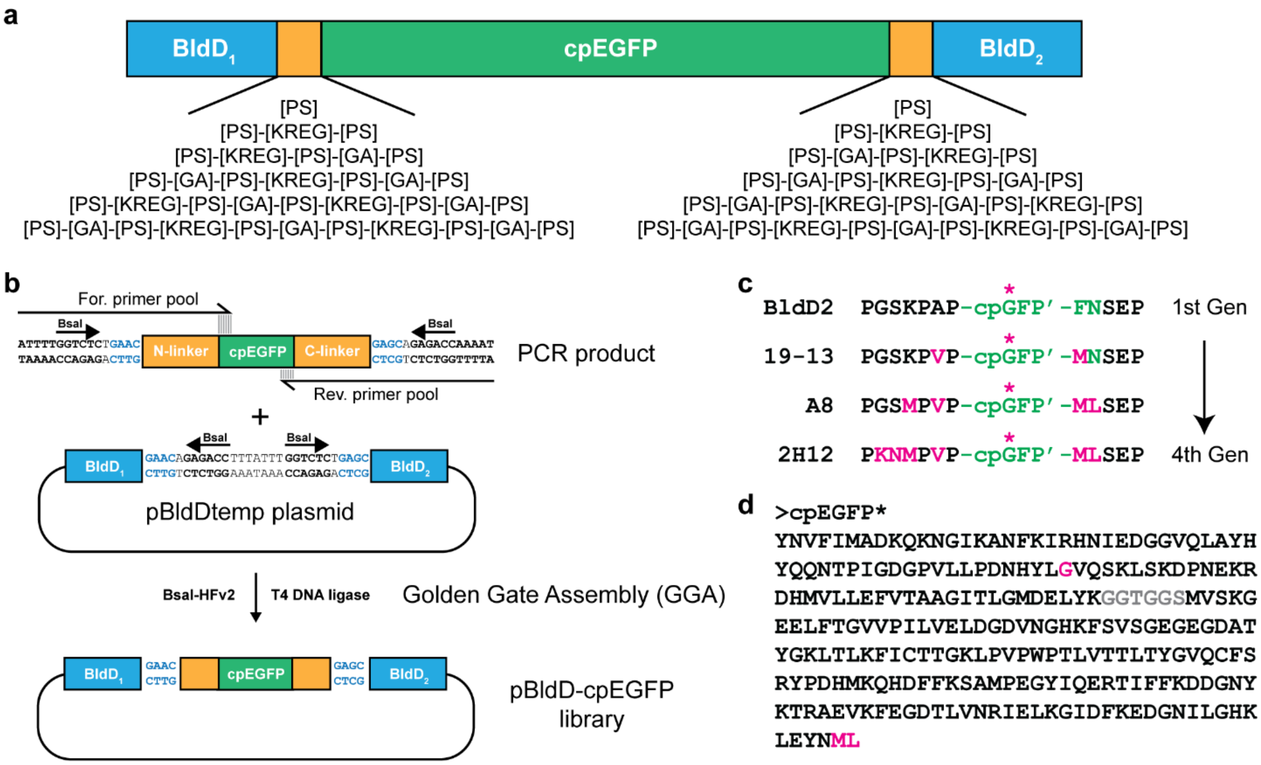

Supplementary Fig. 1: Design and construction of cdGreen biosensor libraries

a, Schematic of first-generation biosensors and all possible designed linker variants N- and C-terminal to cpEGFP. $BldD_1$ and $BldD_2$ indicate the N- and C-terminal BldD protomer, respectively. **b**, Schematic of the primers and cloning strategy used to construct first-generation c-di-GMP biosensor libraries. **c**, Comparison of linker sequences of c-di-GMP biosensor variants from different generations resulting in final cdGreen (a.k.a. 2H12). Residues in magenta indicate amino acid changes compared to the first-generation biosensor (BldD2) and residues in green represent amino acids that are part of the original cpEGFP sequence. The magenta asterisk represents a spontaneously acquired point mutation resulting in a Ser-to-Gly substitution in cpEGFP (see panel d). **d**, Prima ry sequence of evolved cpEGFP used in cdGreen and cdGreen2 biosensors. Residues in magenta differ from parental cpEGFP. The Gly-Ser linker joining the original N- and C-termini of EGFP in the circularly permutated version are highlighted in grey.

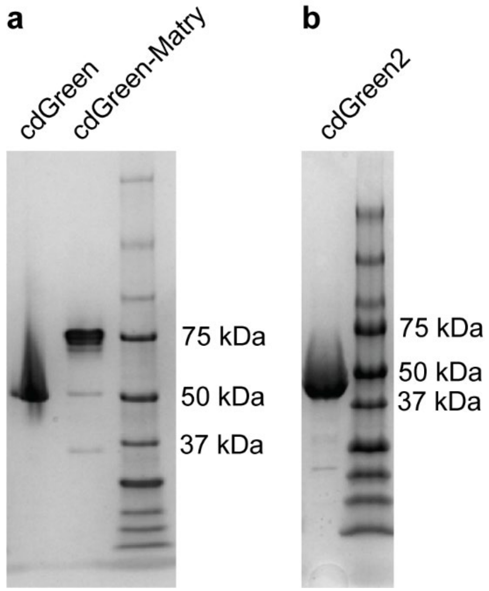

Supplementary Fig. 2: SDS-PAGE analysis with Coomassie staining of purified biosensors

a, SDS-PAGE analysis with Coomassie staining of N-terminally His_6-tagged cdGreen (lane 1) and cdGreen-Matry (lane 2) after NiNTA purification and size exclusion chromatography. A protein standard was run in lane 3 and molecular weights of relevant bands are indicated. **b**, SDS-PAGE analysis with Coomassie staining of N-terminally His_6-tagged cdGreen2 (lane 1) after NiNTA purification and size exclusion chromatography. A protein standard was run in lane 2 and molecular weights of relevant bands are indicated. Source data are provided as a Source Data file.

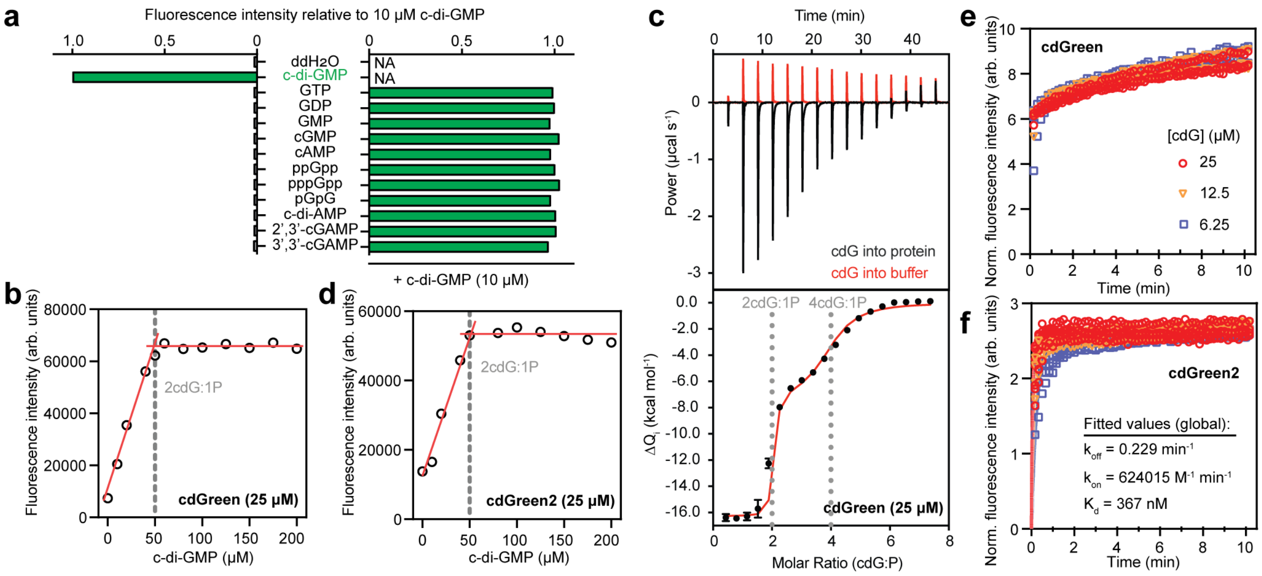

Supplementary Fig. 3: Specificity and stoichiometry of c-di-GMP binding to biosensors

a, Response of purified cdGreen (150 nM) to different nucleotides (10 μM) was measured, with (10 μM) or without additional c-di-GMP. Responses were recorded on a microplate reader and values are 530 nm emission ratios upon excitation with 497 nm and 405 nm, normalized to the value of this signal obtained with cdGreen with 10 μM c-di-GMP. Shown are means of repeated measurements at equilibrium (n=6). **b**, Stoichiometric titration of cdGreen. cdGreen (25 μM) was incubated with varying concentrations of c-di-GMP (see figure) to equilibration and responses were recorded on a microplate reader using emission at 530 nm upon excitation at 497 nm as a readout. Shown are means of repeated measurements at equilibrium (n=6). **c**, Isothermal titration calorimetry (ITC) of cdGreen with c-di-GMP. For details, see Methods and the figure. **d**, Stoichiometric titration of cdGreen2 with c-di-GMP. Experiments were carried out as described for cdGreen in panel b. Shown are means of repeated measurements at equilibrium (n=6). **e**, Association kinetics of cdGreen (for details, see Methods). Values represent 530 nm emission ratios upon excitation with 497 nm and 405 nm. Shown are individual data points from n=3 replicates. Note that GraphPad Prism 9 failed to globally fit kinetic parameters based on these data. **f**, Association kinetics of cdGreen2 (for details, see Methods). Values represent 530 nm emission ratios upon excitation with 497 nm and 405 nm. Shown are individual data points from n=3 replicates. Different c-di-GMP concentrations used are as in panel e. arb. units, arbitrary units; cdG, c-di-GMP. Source data are provided as a Source Data file.

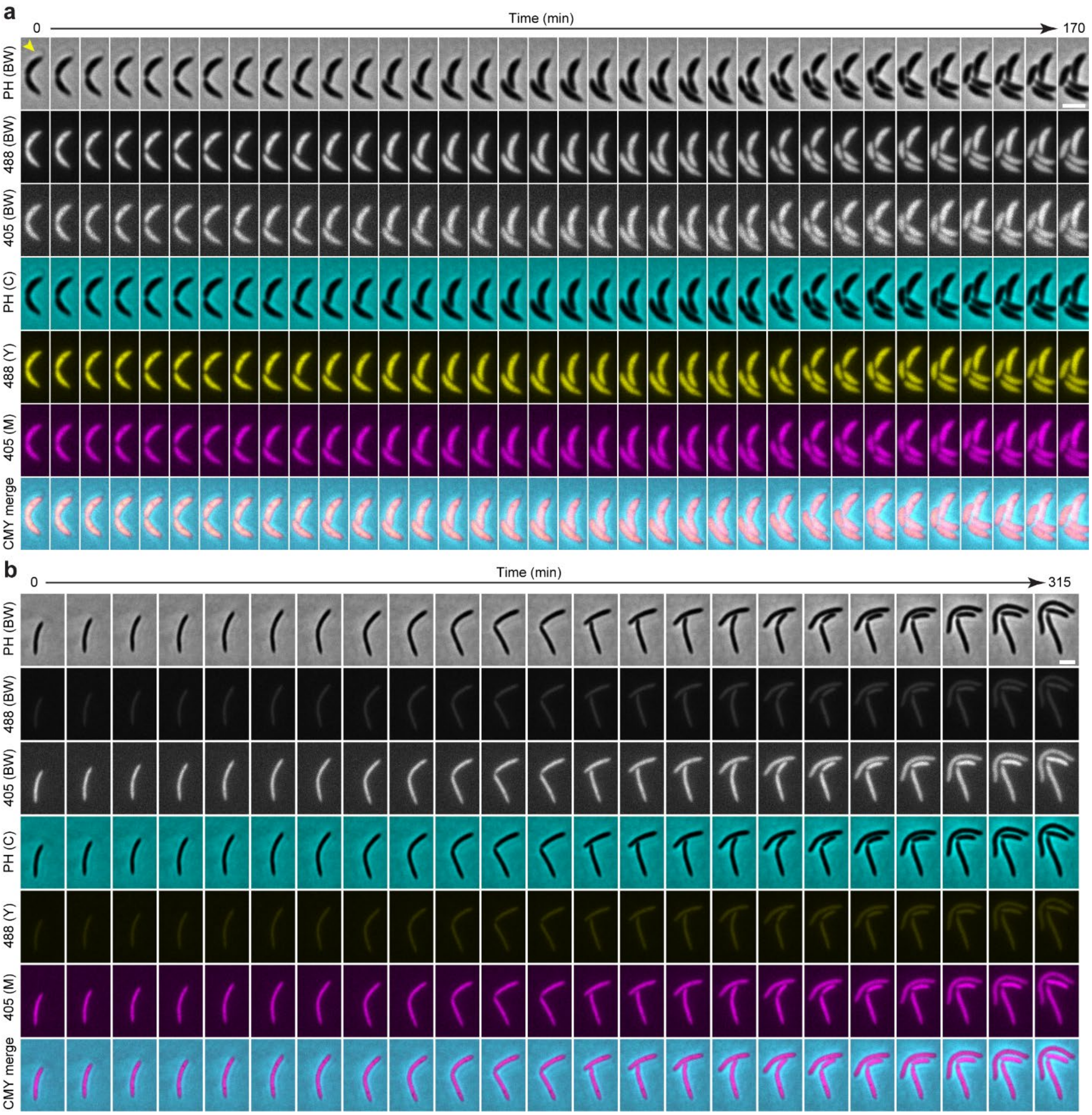

Supplementary Fig. 4: *In vivo* performance of biosensor cdGreen in *C. crescentus*

a, Time-lapse series (5-min intervals) of strain NA1000 carrying plasmid pQFmcs-2H12. Individual channels in black/white (BW) or pseudo-colors (C, M, Y) are shown as well as an overlay of the three pseudo-colored channels. The scale bar indicates 2 µm. The yellow arrow indicates the stalked pole. **b**, Time-lapse series (15-min intervals) of strain NA1000 rcdG^0 carrying plasmid pQFmcs-2H12. Individual channels in black/white (BW) or pseudo-colors (C, M, Y) are shown as well as an overlay of the three pseudo-colored channels. The scale bar indicates 2 µm.

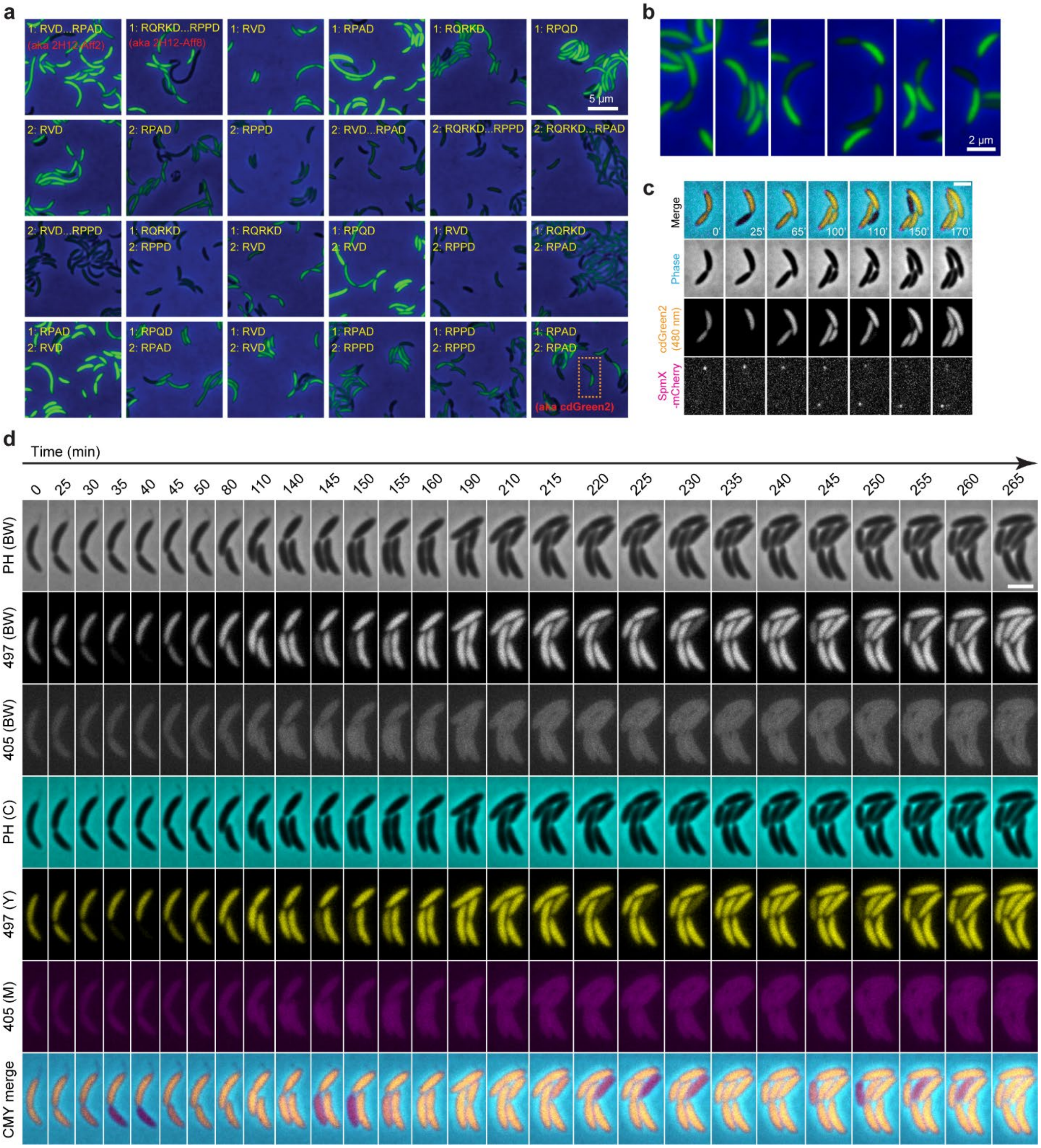

Supplementary Fig. 5: Screening and isolation of biosensor cdGreen2

a, Microscopy snapshots of *C. crescentus* strains carrying different 2H12 derivatives (see Methods for details). Variants of c-di-GMP binding motifs in the N-terminal BldD CTD (1) and/or the C-terminal BldD CTD (2) are indicated in the top left corner of each panel. A single late predivisional showing asymmetric c-di-GMP levels is highlighted by an orange box (variant "1: RPAD, 2: RPAD" aka cdGreen2). **b**, More examples of late predivisional cells with asymmetric c-di-GMP levels in the "1: RPAD, 2: RPAD" sample from the same screening setup shown in panel a. **c**, Time-lapse series of a NA1000 strain chromosomally expressing mCherry-tagged SpmX, serving as a ST cell-specific marker, and carrying plasmid pQFmcs-2H12.D11. Time stamps are in minutes. The scale bar represents 2 µm. **d**, Time-lapse series of strain

NA1000 carrying plasmid pQFmcs-2H12.D11. Individual channels in black/white (BW) or pseudo-colors (C, M, Y) are shown as well as an overlay of the three pseudo-colored channels. The scale bar indicates 2 µm. Note that this time-lapse series is part of the series shown in Fig. 3c.

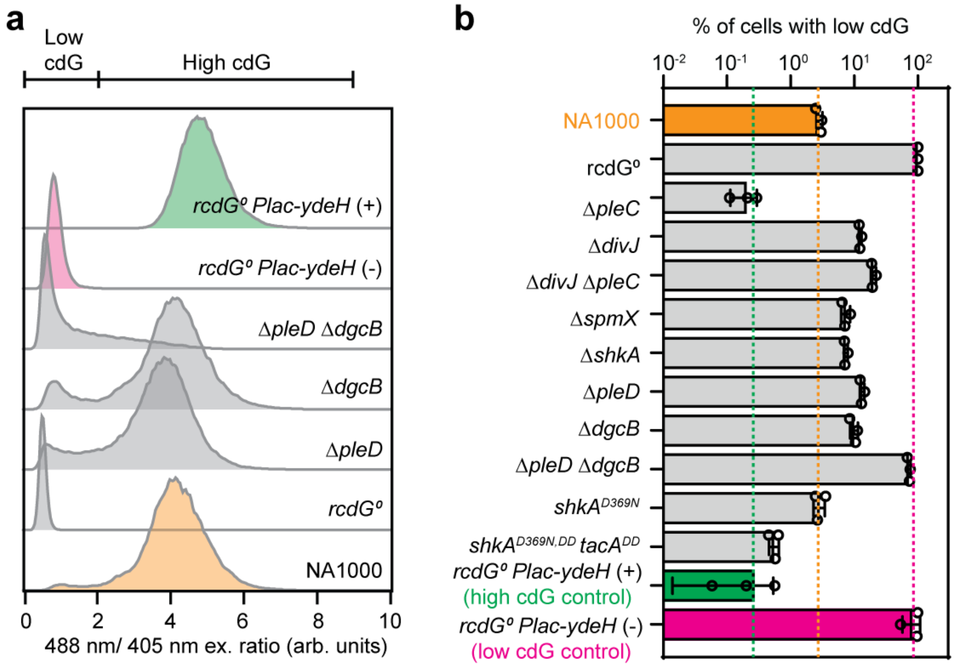

Supplementary Fig. 6: C-di-GMP quantification by flow cytometry in *C. crescentus*

a, Flow cytometry analysis of indicated strains. The rcdG^0 *Plac-ydeH* strain lacks all endogenous diguanylate cyclases and phosphodiesterases, but harbors a chromosomal, IPTG-inducible copy of the heterologous diguanylate cyclase DgcZ from *E. coli*; no IPTG (-), 1 mM IPTG (+). For each event measured, the 488 nm/ 405 nm ratio is given. On top, the gates used for classification as cells with low or high c-di-GMP (see panel b) are indicated. **b**, Quantification of fractions of cells with low c-di-GMP levels (as defined by the gates shown in panel a) for indicated strains. Shown are means and standard deviations of three biological replicates per strain and condition. arb. units, arbitrary units; cdG, c-di-GMP. Source data are provided as a Source Data file.

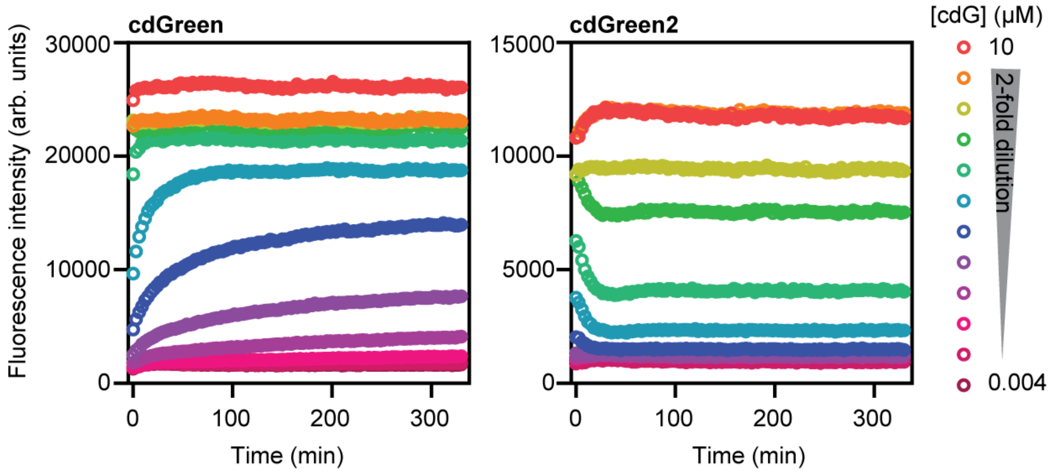

Supplementary Fig. 7: Equilibration of c-di-GMP/biosensor complexes for *in vitro* experiments

Indicated concentrations of c-di-GMP were added to 150 nM biosensor in sensor buffer (see Methods) and the biosensor signal (excitation at 497 nm, emission at 530 nm) was recorded over time at 30°C. arb. units, arbitrary units; cdG, c-di-GMP.

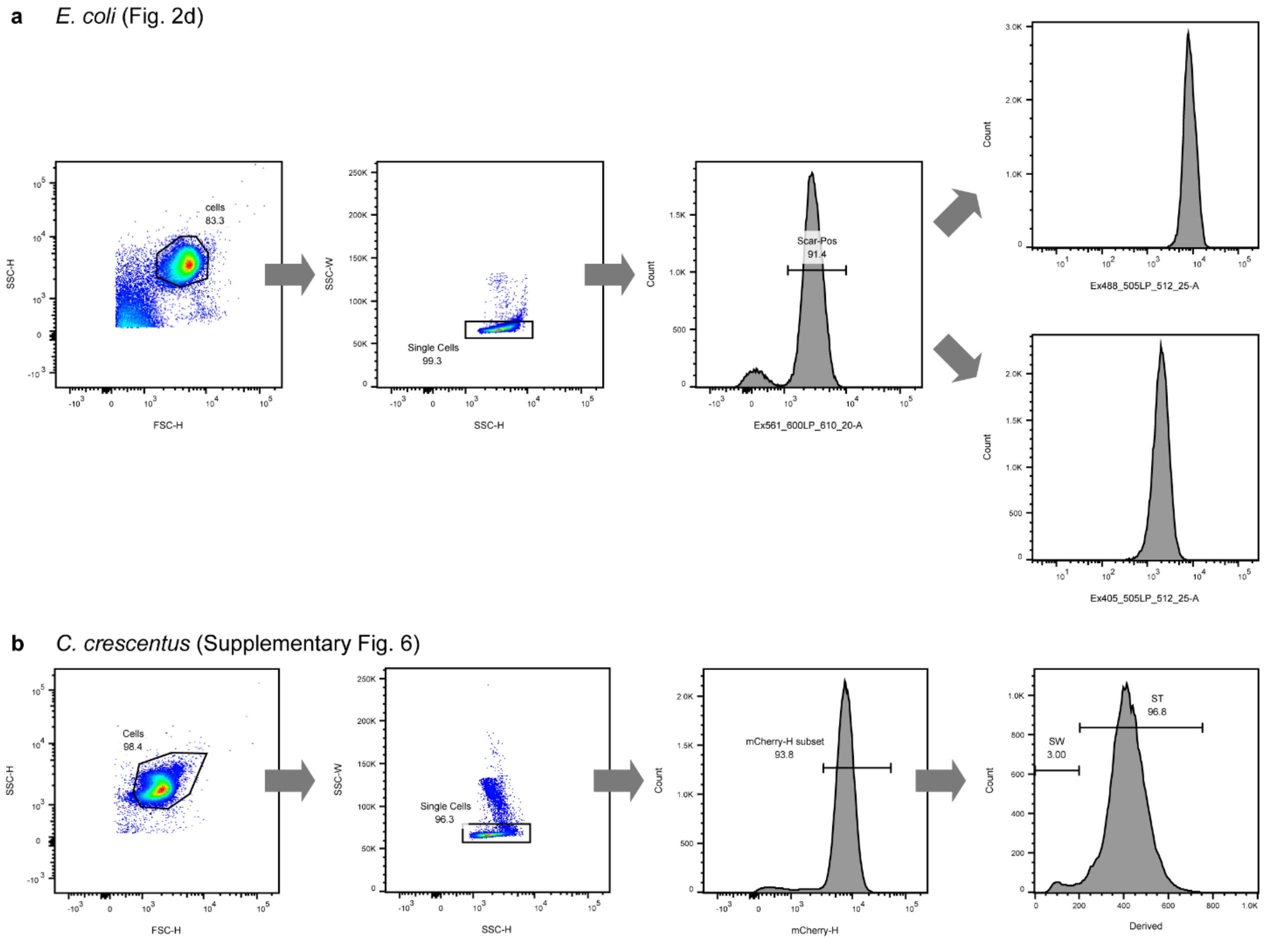

Supplementary Fig. 8: Gating strategy for flow cytometry

Gating strategy for flow cytometry used for *E. coli* (**a**) and *C. crescentus* (**b**).
