# Supplementary material for: A genetically encoded biosensor to monitor dynamic changes of c-di-GMP with high temporal resolution
Source: Nat Commun. 2024 May 9;15:3920. doi: 10.1038/s41467-024-48295-0 (PMC11082216; doi:10.1038/s41467-024-48295-0)
Supplement: Supplementary file 3 — Description of Additional Supplementary Files [file 41467_2024_48295_MOESM3_ESM.pdf]

Description of Additional Supplementary Files

Supplementary Data 1: Oligonucleotides, plasmids, strains and synthetic DNA used in this study Source Data file.

Supplementary Movie 1: High-temporal-resolution imaging of c-di-GMP levels in C. crescentus: https://zenodo.org/records/10965397

Time-lapse movie of C. crescentus NA1000 cells carrying pQFmcs-2H12.D11-scarREF imaged in 20-sec intervals over ~3 hours at 40 frames per seconds. The right panel shows the phase contrast channel; the left panel shows an overlay of the cdGreen2 FITC/FITC signal pseudo-colored in yellow, the mScarlet-I signal pseudo-colored in magenta and the phase contrast signal pseudo-colored in cyan.
