# Supplementary material for: A genetically encoded biosensor to monitor dynamic changes of c-di-GMP with high temporal resolution
Source: Nat Commun. 2024 May 9;15:3920. doi: 10.1038/s41467-024-48295-0 (PMC11082216; doi:10.1038/s41467-024-48295-0)
Supplement: Supplementary file 6 — Reporting Summary [file 41467_2024_48295_MOESM6_ESM.pdf]

nature portfolio

Corresponding author(s): Urs Jenal
Andreas Kaczmarczyk

Last updated by author(s): Apr 12, 2024

Reporting Summary

Nature Portfolio wishes to improve the reproducibility of the work that we publish. This form provides structure for consistency and transparency in reporting. For further information on Nature Portfolio policies, see our Editorial Policies and the Editorial Policy Checklist.

Statistics

For all statistical analyses, confirm that the following items are present in the figure legend, table legend, main text, or Methods section.

n/a	Confirmed	
☐	☒	The exact sample size (*n*) for each experimental group/condition, given as a discrete number and unit of measurement
☐	☒	A statement on whether measurements were taken from distinct samples or whether the same sample was measured repeatedly
☒	☐	The statistical test(s) used AND whether they are one- or two-sided<br>*Only common tests should be described solely by name; describe more complex techniques in the Methods section.*
☒	☐	A description of all covariates tested
☒	☐	A description of any assumptions or corrections, such as tests of normality and adjustment for multiple comparisons
☐	☒	A full description of the statistical parameters including central tendency (e.g. means) or other basic estimates (e.g. regression coefficient) AND variation (e.g. standard deviation) or associated estimates of uncertainty (e.g. confidence intervals)
☒	☐	For null hypothesis testing, the test statistic (e.g. *F*, *t*, *r*) with confidence intervals, effect sizes, degrees of freedom and *P* value noted<br>*Give P values as exact values whenever suitable.*
☒	☐	For Bayesian analysis, information on the choice of priors and Markov chain Monte Carlo settings
☒	☐	For hierarchical and complex designs, identification of the appropriate level for tests and full reporting of outcomes
☒	☐	Estimates of effect sizes (e.g. Cohen's *d*, Pearson's *r*), indicating how they were calculated

Our web collection on statistics for biologists contains articles on many of the points above.

Software and code

Policy information about availability of computer code

Data collection	FACS/flow cytometry data were acquired with BD FACSDiva 8.0.1. Microscopy data were acquired with softWoRx 6.0 (Deltavision) or NIS Elements AR v.4.51 (Ti2). Plate reader data were acquired with BioTek Gen5. MO.Control (Nanotemper) was used for ITC.
Data analysis	FACS and flow cytometry data were analyzed using FlowJo v10. Microscopy images were prepared in OMERO (University of Dundee & Open Microscopy Environment) and quantified in Fiji using the MicrobeJ plugin (v5). Affinimeter was used to analyse ITC data. Numerical data were collected, filtered and sorted in Excel 2016 (Microsoft) and analysed in GraphPad Prism v9. Time lapse microscopy data were processed and analysed using DeLTA 2.0, Napari (https://zenodo.org/record/3555620) and custom Python code deposited on Github: https://github.com/simonvanvliet/cdg_dynamics_caulobacter.git and Zenodo: https://doi.org/10.5281/zenodo.10900331

For manuscripts utilizing custom algorithms or software that are central to the research but not yet described in published literature, software must be made available to editors and reviewers. We strongly encourage code deposition in a community repository (e.g. GitHub). See the Nature Portfolio guidelines for submitting code & software for further information.

Data

Policy information about availability of data

All manuscripts must include a data availability statement. This statement should provide the following information, where applicable:

- Accession codes, unique identifiers, or web links for publicly available datasets
- A description of any restrictions on data availability
- For clinical datasets or third party data, please ensure that the statement adheres to our policy

The datasets generated during and/or analysed during the current study are available in the Source Data file provided with the paper or from Zenodo: https://zenodo.org/records/10950147 and https://zenodo.org/records/10965397. Select plasmids, in particular those encoding c-di-GMP biosensors, will be made available through the public plasmid repository Addgene (https://www.addgene.org/Urs_Jenal/) upon publication. Other unique biological material is available from the corresponding authors on reasonable request.

Research involving human participants, their data, or biological material

Policy information about studies with human participants or human data. See also policy information about sex, gender (identity/presentation), and sexual orientation and race, ethnicity and racism.

Reporting on sex and gender	n/a
Reporting on race, ethnicity, or other socially relevant groupings	n/a
Population characteristics	n/a
Recruitment	n/a
Ethics oversight	n/a

Note that full information on the approval of the study protocol must also be provided in the manuscript.

Field-specific reporting

Please select the one below that is the best fit for your research. If you are not sure, read the appropriate sections before making your selection.

☒ Life sciences ☐ Behavioural & social sciences ☐ Ecological, evolutionary & environmental sciences

For a reference copy of the document with all sections, see nature.com/documents/nr-reporting-summary-flat.pdf

Life sciences study design

All studies must disclose on these points even when the disclosure is negative.

Sample size	No calculations of sample sizes were performed. Sample sizes were such that they were representative and were usually performed in triplicate, as is standard practice. For flow cytomertry, 50'000 events were usually acquired in the SSC-H/SSC-W gate.
Data exclusions	No.
Replication	Typically, experiments were performed in triplicate, unless they were part of the screening/selection pipeline for c-di-GMP biosensors, in which case the experiment was performed once (final biosensors were eventually characterized in more detail with replicate experiment). Time lapse microscopy experiments were performed in duplicate. All attempts at replication were successful.
Randomization	No. No systematic errors were expected to be introduced by covariates in this study and therefore covariates were not controlled for.
Blinding	Analysis of time-lapse microscopy movies was blind (data recorded by A. Ka, and analysed by S.v.V).

Reporting for specific materials, systems and methods

We require information from authors about some types of materials, experimental systems and methods used in many studies. Here, indicate whether each material, system or method listed is relevant to your study. If you are not sure if a list item applies to your research, read the appropriate section before selecting a response.

Materials & experimental systems

n/a	Involved in the study
☒	☐ Antibodies
☒	☐ Eukaryotic cell lines
☒	☐ Palaeontology and archaeology
☒	☐ Animals and other organisms
☒	☐ Clinical data
☒	☐ Dual use research of concern
☒	☐ Plants

Methods

n/a	Involved in the study
☒	☐ ChIP-seq
☐	☒ Flow cytometry
☒	☐ MRI-based neuroimaging

Plants

Seed stocks

Report on the source of all seed stocks or other plant material used. If applicable, state the seed stock centre and catalogue number. If plant specimens were collected from the field, describe the collection location, date and sampling procedures.

Novel plant genotypes

Describe the methods by which all novel plant genotypes were produced. This includes those generated by transgenic approaches, gene editing, chemical/radiation-based mutagenesis and hybridization. For transgenic lines, describe the transformation method, the number of independent lines analyzed and the generation upon which experiments were performed. For gene-edited lines, describe the editor used, the endogenous sequence targeted for editing, the targeting guide RNA sequence (if applicable) and how the editor was applied.

Authentication

Describe any authentication procedures for each seed stock used or novel genotype generated. Describe any experiments used to assess the effect of a mutation and, where applicable, how potential secondary effects (e.g. second site T-DNA insertions, mosiacism, off-target gene editing) were examined.

Flow Cytometry

Plots

Confirm that:

☐ The axis labels state the marker and fluorochrome used (e.g. CD4-FITC).

☐ The axis scales are clearly visible. Include numbers along axes only for bottom left plot of group (a 'group' is an analysis of identical markers).

☐ All plots are contour plots with outliers or pseudocolor plots.

☐ A numerical value for number of cells or percentage (with statistics) is provided.

Methodology

Sample preparation	Aliquots of bacterial cultures were diluted in PBS or ddH2O. Details are described in the Methods section.
Instrument	BD FACSAria III; BD LSRFortessa Cell Analyzer
Software	BD FACSDiva 8.0.1 (BD Biosciences)
Cell population abundance	Sorts were performed to enrich for functional biosensors as described in the manuscript. Post-sorts were not done systematically. For characterization of biosensors, clonal bacterial cultures were used and typically 50'000 events were acquired.
Gating strategy	1. FSC-H/SSC-H (for cells). 2. SSC-H/SSC-W (for singlets). Optional: 3. mCherry-H histogram (for mScarlet-I-positive cells). The gating strategies are exemplified in Supplementary Figure 8.

☒ Tick this box to confirm that a figure exemplifying the gating strategy is provided in the Supplementary Information.
